# Open and Closed Endotracheal Suctioning and Arterial Blood Gas Values: A Single-Blind Crossover Randomized Clinical Trial

**DOI:** 10.1155/2015/470842

**Published:** 2015-09-03

**Authors:** Azam Faraji, Alireza Khatony, Gholamreza Moradi, Alireza Abdi, Mansour Rezaei

**Affiliations:** ^1^Kermanshah School of Nursing and Midwifery, Kermanshah University of Medical Sciences, Kermanshah, Iran; ^2^Imam Ali Hospital, Kermanshah University of Medical Sciences, Kermanshah, Iran; ^3^School of Health, Kermanshah University of Medical Sciences, Kermanshah, Iran

## Abstract

*Aim*. This study was aimed at comparing the effects of the open and closed suctioning techniques on the arterial blood gas values in patients undergoing open-heart surgery. *Methods*. In a clinical trial, we recruited 42 patients after open-heart surgery in an educational hospital. Each patient randomly underwent both open and closed suctioning. ABGs, PaO_2_, SaO_2_, PaCO_2_, were analyzed before and one, five, and fifteen minutes after each suctioning episode. *Results*. At first the pressure of oxygen in arterial blood increased; however, this increase in the open technique was greater than that of the closed system (*P* < 0.001). The pressure of oxygen decreased five and fifteen minutes after both suctioning techniques (*P* < 0.05). The trends of carbon dioxide variations after the open and closed techniques were upward and downward, respectively. Moreover, the decrease in the level of oxygen saturation five and fifteen minutes after the open suctioning was greater than that of the closed suctioning technique (*P* < 0.05).  *Conclusion*. Arterial blood gas disturbances in the closed suctioning technique were less than those of the open technique. Therefore, to eliminate the unwanted effects of endotracheal suctioning on the arterial blood gases, the closed suctioning technique is recommended.

## 1. Background

Clearance of airway secretions is a normal physiological process needed for the preservation of airway patency and the prevention of respiratory tract infection. Impaired clearance of airway secretions can result in atelectasis and pneumonia and may contribute to respiratory failure [1]. Intubated patients in intensive care units (ICUs) deal with many problems in adequately coughing up secretions [2], which consequently result in the obstruction of tube lumen, increased respiratory work, pulmonary infections, alteration of the heart rate, hypoxemia, and ventilator-associated pneumonia (VAP) [3]. To reduce these complications, endotracheal suctioning (ES) technique is a common procedure that is performed 8–17 times a day on the patients in ICU [4]. By removing the pulmonary secretions, ES can help to establish and maintain gas exchange, adequate oxygenation, and alveolar ventilation [5, 6].

Nowadays, two systems are available to perform ES: the single-use, open suction (OS) and the multiple-use, closed suction (CS). OS requires disconnection from the ventilator during ES, which is not necessary when using CS. Moreover, in contrast to OS, the CS catheter can remain connected to the patient for 24 hrs according to the manufacturer and thus can be used for multiple ES procedures [3]. However, regarding the selection of one of the ES methods (OS or CS), there are some ambiguities because each of them has many advantages and some disadvantages, and there are controversial results about the superiority of one method over the other. In CS there are some benefits like lower hemodynamic impairment, less possibility of aspiration, and decreasing environmental contamination from respiratory microorganisms compared to OS and some flaws such as higher cost, less efficacy to remove secretions, and causing unpredictable high level of interstice positive end-expiratory pressure [7]. Vonberg et al. [8] reported no significant advantages for selecting ES systems (OS and CS) to prevent ventilation-associated pneumonia. Jongerden et al. [9] stated there is controversy regarding the efficacy of these systems (OS versus CS) in reducing infections, oxyhemodynamic changes, and length time on mechanical ventilation as well as the period of hospitalization. In recent decades, the closed suction system has been gaining popularity in the developed countries. In the United States, for example, this system is used in 58% of ICUs, while the open system is used exclusively in only 4% of the centers, but there is no evidence to support CS superiority over OS [3]. The effects of OS and CS on the arterial blood gases (ABGs) have been investigated in several studies, with controversial and partly paradoxical results. Cereda et al. [10] reported that, in the OS technique, O_2_ saturation (SaO_2_) decreased significantly but this change was not different compared with CS technique. Lasocki et al. [11] also compared the effects of OS and CS techniques on gas exchange in patients with acute lung injury and found that PaO_2_ did not change significantly in the CS technique.

In a study by Jongerden et al. [9], there was no significant difference between heart rate (HR), mean arterial pressure (MAP), and peripheral oxygen saturation (Spo2) in patients undergoing CS and OS. In the study conducted by Taheri et al. [12], hemodynamic status (RR, Spo2) was better in CS then in OS, and respiratory complications were less. Also, in other studies, Hoellering et al. [13], Kuriyama et al. [14], and Sakuramoto et al. [15], paradoxical results were found about the superiority of one method (OS versus CS) over the other in terms of respiratory parameters in intubated patients.

Since ES is a potentially harmful procedure, if performed inappropriately or incorrectly, it might result in life-threatening complications such as bleeding, infection, cardiovascular instability, and elevated intracranial pressure and may also cause lesions in the tracheal mucosa of the ICU patients [2, 16]. These side effects can be far more serious in cardiac surgical patients [17]; therefore, more studies are needed to select the best method of ES. Given the controversies about the use of ES methods in the aforementioned studies, low level of ES performance in Iranian nurses [18], and the serious situation of the patients undergoing open-heart surgery (OHS), this study was conducted to compare the effects of OS and CS techniques on the ABG values in patients undergoing OHS.

## 2. Methods

### 2.1. Design

This study was a single-blind crossover randomized clinical trial which was conducted over 6 months between April and September 2010. The population included all patients admitted to Intensive Care Units (ICUs) of Imam Ali Hospital in Kermanshah-west of Iran. The study population was selected via convenience sampling technique and included the patients who underwent OHS and were hospitalized in the ICU of Imam Ali hospital affiliated to KUMS. The sample consisted of the patients who had an arterial line; therefore, it was not necessary to frequently insert needle into the arterial vascular system to obtain arterial blood samples for ABG checking. The sample size was calculated according to the standard deviation of PaO_2_ before and after suction in similar study [11], confidence level of 95%, and the test power of 90%. Thus, 21 patients were estimated; however 42 patients were recruited to cover all objectives. All eligible patients were recruited during the study period until the sample size was fulfilled. Hence, the participants were allocated to either control or experimental group by random number tables.

The inclusion criteria, which were also reported in other studies [19, 20] and designed to reduce the possibility of attrition, comprised undergoing coronary artery by-pass grafting, receiving mechanical ventilation through endotracheal tube, having stable hemodynamic parameters (blood pressure, mean arterial pressure, and heart rate), having an ejection fraction more than 50%, having a chest tube output of less than 100 milliliters per hour, having adequate tissue perfusion (with checking capillary refill time less than 3 seconds) and urinary output (by taking clinical history), having a body temperature of 35.5 to 38.5, having normal hematocrit (36–50%) and hemoglobin values (12–17 g/dL), having normal PaO_2_ (80–100 mmHg) and PaCO_2_ (35–45 mmHg) values, being 30–75 years old, and giving informed consent to participate in the study. Patients with instability in hemodynamic parameters, renal failure, and pulmonary diseases such as emphysema and asthma as well as chronic cigarette smokers were excluded from the study. The first author generated the allocation sequence, enrolled the participants, and divided them either to the OS or to CS technique.

The data collection instrument was a designed sheet which consisted of two sections, including a demographic questionnaire and a data sheet for documenting the ventilator setting and ABG values (PaO_2_, PaCO_2_, and SaO_2_). ABGs were analyzed using the Nova-biomedical blood gas analyzer, made in the United States. To assure the reliability of the analyzer, we calibrated the analyzer before each analysis. The suctioning machine was the portable Medola-dominant machine, made in Switzerland. Data were collected by taking ABG from the patients before and 1, 5, and 15 minutes after each suctioning session and observing the examination results, which were eventually written in the data sheets.

### 2.2. Intervention

Before going to the operation room for OHS, the eligible patients were recruited and registered in the study. The symptoms needed for suctioning, which were diagnosed by the staff nurse in the ICU ward, were excessive secretions, abnormal lung sounds, and decreased SaO_2_. Suctioning was performed by the researcher when the staff nurse called it as required.

We randomly assigned each patient to receive either the OS or CS. If the patient received the OS technique at the first suctioning episode, he was planned to receive the CS technique at the next episode, and vice versa. Therefore, in our study each patient received two episodes of suctioning: one episode of OS and one episode of CS. To eliminate the effects of the first episode, we included at least a 90-minute washout interval between the two episodes; when the patients needed suctioning, it was repeated again in another way and if the patients needed suctioning during the washout interval, they were excluded from the study.

**Figure 1:**
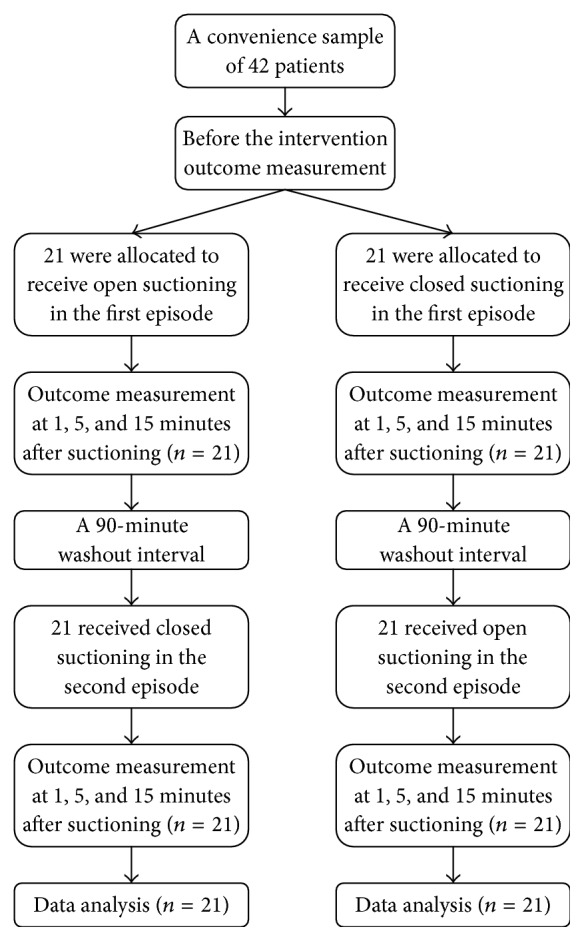
Flow diagram of the study process.

Before the first suctioning episode, we obtained an arterial blood sample and analyzed it for ABGs. The values of this ABG analysis were considered as the basic values (henceforth referred to as T0). Accordingly, one, five, and fifteen minutes after each episode of suctioning, we again obtained arterial blood samples and determined ABGs for each sample (henceforth, we refer to these three postsuctioning measurement time points as T1, T2, and T3). Two minutes before and after each suctioning episode, all the patients were hyperventilated with 100% oxygen.  Figure 1 illustrates the study process.

The suctioning time in each episode was around 10–15 seconds, which was measured by a chronometer. The suctioning techniques were done with respect to the acceptable standards [2], including the size of the suctioning catheter which was half the internal diameter of the endotracheal tube. To apply the open technique, we began with hyperoxygenating the patients and disconnecting them from ventilator. Thereafter, with the suctioning machine turned off, the suctioning catheter was inserted into the endotracheal tube. Then, the machine was turned on and the researcher began to remove the catheter by twisting it around his own thumb and index finger. In the OS technique, safety equipment items such as sterile gloves, protective glasses, and face mask were necessary to prevent contamination with patient's bodily secretions.

In the CS technique, without disconnecting the patients from ventilator, the suctioning catheter (Trach Care, Ballard Medical Products, Draper, Utah, made in USA) was connected to the Y-shaped piece and suctioning was started. The Y-shaped piece was already located between the endotracheal tube and the ventilator tube. In the CS technique, it is not necessary to wear sterile gloves; however, the researcher wore nonsterile gloves to prevent contamination with patients' bodily secretions. To place the closed suction or to separate it, the patients were hyperventilated by 100% oxygen two minutes before and after changing the suction system, and the replacement process was done by a staff nurse, which lasted less than 20 seconds.

### 2.3. Data Analysis

We employed the 16th version of the Statistical Package for Social Sciences (SPSS v.16.0; SPSS Inc., Chicago, IL, USA) for data management and analysis. First, we used Kolmogorov-Smirnov test to test the normality of data. Because the study variables were distributed nonnormally, we used the nonparametric tests. To compare the ABG values between the two techniques at different measurement times, we used the Wilcoxon ranked test and Friedman's ANOVA by rank. Wilcoxon test is analogues to the parametric paired *t*-test [21], which was used in this study to assess the difference between ABG parameters of one group in two different suctioning methods. Friedman's ANOVA by rank, which is a nonparametric test similar to repeated measures ANOVA in parametric tests, was used to test the differences in the median value of an ordinal, interval, or ratio variable with repeated measures of the dependent variable [21], and, in this study, it was used to compare the ABG values at different measurement times following each suctioning episode. Since the patients served as their own control, the comparisons were made only between the ABG values at different times. Moreover, there were not any confounding factors because of the same patients in various ABG measurements. *P* values less than 0.05 were considered as significant level.

## 3. Results

From forty-two patients who participated in the study, 64.3% (27 patients) were male. The age of patients was between 30 and 75 and the mean and standard deviation (SD) were 62.61 ± 9.48 (Table 1). The size of the endotracheal tube was eight French in 64.3% (27 people) of patients and 7.5 in the rest of the patients.

### 3.1. PaO_2_

**Figure 2:**
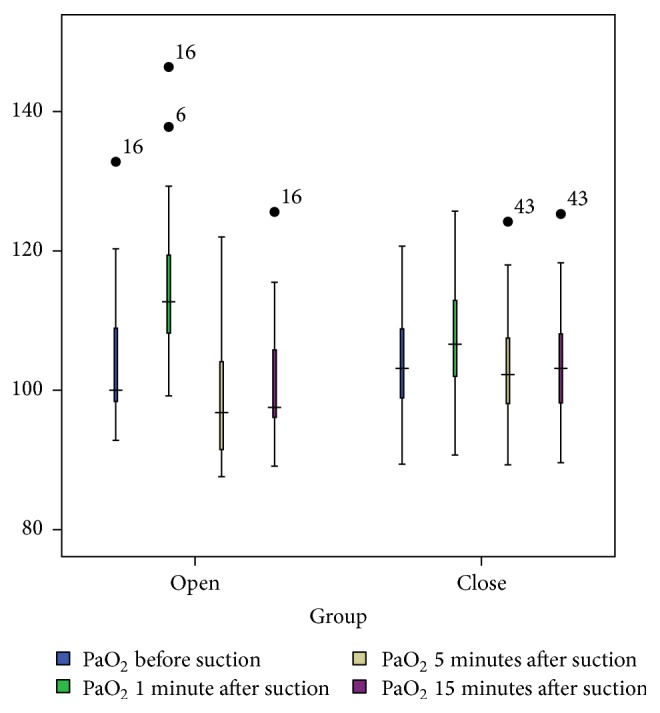
PaO_2_ levels (mmHg) in the OS and CS techniques at four measurement times.

The results of Wilcoxon test showed no statistically significant difference between the OS and CS techniques in terms of the level of PaO_2_ at T0. However, this difference was statistically significant at T1–T3 (*P* < 0.001; see Table 2). On the other hand, the results of Friedman's rank test showed that PaO_2_ level across the four measurement time points, that is, T0–T3, differed significantly in both the OS and the CS techniques (*P* < 0.001; see Table 2). However, the trend of variations of PaO_2_ in the OS technique from T0 to T3 was greater than that of the CS technique (Figure 2).

### 3.2. PaCO_2_

**Figure 3:**
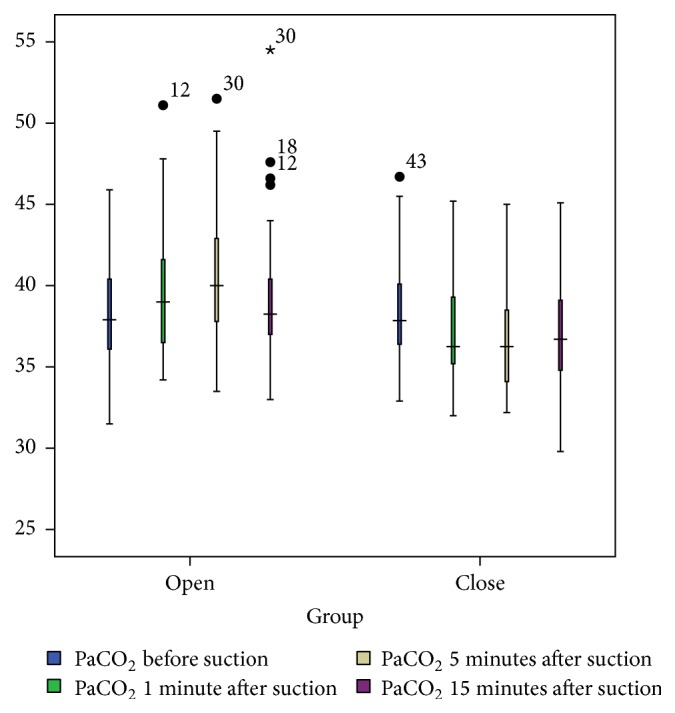
PaCO_2_ levels (mmHg) in the OS and CS techniques at four measurement times.

The results of the Wilcoxon test showed that PaCO_2_ level before the OS technique did not differ significantly with PaCO_2_ level before the CS technique. However, the differences between the two techniques at T1, T2, and T3 were statistically significant (*P* < 0.001; Table 2). The results of Friedman's rank test again showed a significant difference in PaCO_2_ level from T0 to T3 in both the OS and the CS techniques (*P* < 0.001; Table 2). However, the trend of variations of PaCO_2_ in the OS technique was upward, while the trend in the CS technique was downward (Figure 3).

### 3.3. SaO_2_

**Figure 4:**
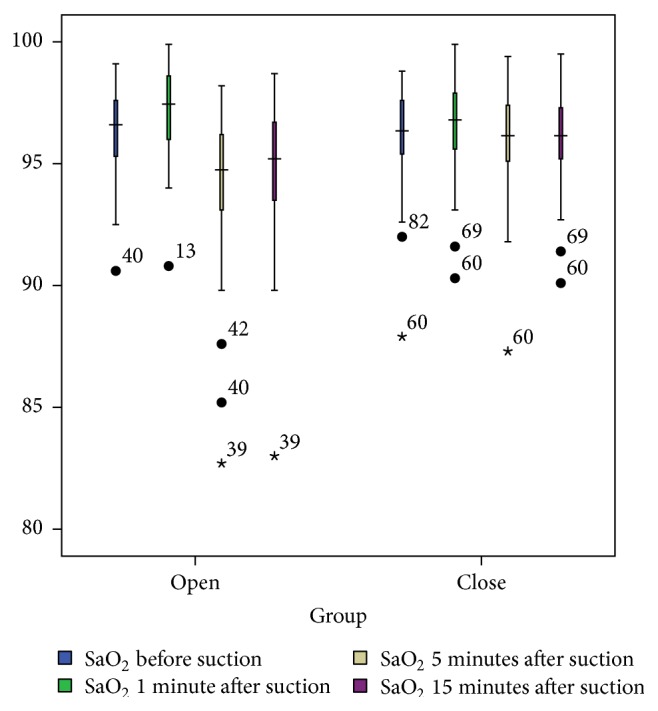
SaO_2_ levels (%) in the OS and CS techniques at four measurement times.

Based on the results of the Wilcoxon test, there was no statistically significant difference between the OS and the CS techniques in terms of SaO_2_ level at T0. However, the results of the test showed statistically significant differences between the two suctioning techniques at T1, T2, and T3 (*P* < 0.005; Table 2). On the other hand, the results of Friedman's rank test showed that SaO_2_ level in both suctioning techniques from T0 to T3 differed significantly (*P* < 0.001; Table 2). However, the trend of variations of SaO_2_ in the OS technique was greater than that of the CS technique (Figure 4).

## 4. Discussion

The study findings showed that PaO_2_ increment one minute after the OS technique was greater than the increment one minute after the CS technique; however, PaO_2_ decreased five and fifteen minutes after both the OS and CS techniques. Compared to the CS technique, this decrease was greater in the OS technique. Generally, the trend of variations in ABG values in the OS technique was greater than that of the CS technique. In line with our study, the results of Liu et al. [22] showed that PaO_2_ values in the open suction group immediately after suction were significantly lower than PaO_2_ values before suction, and PaO_2_ value two minutes after suction dropped. PaO_2_ values in the closed suction also dropped, but there were no significant differences compared with the values before suction. Lee et al. [23] also found that PaO_2_ decrease in the open technique was greater than that of the closed technique. Lasocki et al. [11] also reported that, compared to the closed technique, the open technique caused 18% more decrease in PaO_2_. However, Cereda et al. [10] did not observe any significant differences in blood gas parameters before and after either procedure. Moreover, in a meta-analysis by Jongerden et al. [3], there was not any difference between OS and CS systems in terms of PaO_2_, and both systems were reported to be equally safe.

The difference in the results of the abovementioned studies could be attributed to the differences in the methodologies and size of samples. It is believed that, in ICU patients who are dependent on ventilator, the most significant loss in the lung volume during suctioning occurs primarily during disconnection of ventilator. Hence, OS technique results in a greater lung volume loss when compared with CS [24]. CS can also maintain continuous ventilation and positive end-expiratory pressure (PEEP) to avoid or reduce alveolar atrophy and to stabilize PaO_2_ to avoid impairment of gas exchange and suction-induced hypoxemia [22]. Özden and Görgülü [25] also recommended using CS technique for patients undergoing open-heart surgery because it reduces variation in HR, MAP, PaO_2_, PaCO_2_, and SaO_2_, to prevent related complications and to enhance the patients' safety. In the view of other researchers, CS is preferred for intubated patients, especially for patients with significant lung disease and those who require high positive end-expiratory pressures in order to avoid alveolar derecruitment and hypoxemia exacerbation during endotracheal tube suctioning [24]. It appears that fundamental efforts are necessary to be made in order to prevent postoperative atelectasis and consequently its inverse effects on PaO_2_ and impaired gas exchange in OHS patients [26]; so, CS can be considered as a technique of choice.

The findings also revealed that after the OS technique PaCO_2_ had an upward slope, whereas PaCO_2_ slope after the CS technique was downward. In other words, while PaCO_2_ increased after the open technique, it decreased after the closed technique. In line with our study, Nazmiyeh et al. [27] also reported that, after the open and closed techniques, PaCO_2_ decreased by 3% and 4%, respectively. The findings of Lasocki et al. [11] showed that OS system induced 8% increase in PaCO_2_, but in CS no change was observed in PaCO_2_ compared with baseline values. But, in Özden and Görgülü [25] and Uğraş and Aksoy [28] studies, there was no significant difference between OS and CS in terms of PaCO_2_.

The difference in the above results can be due to differences in methodologies (such as suctioning duration and measurement time) and samples. Increase of PaCO_2_ in OS technique stimulates chemoreceptors in the aorta and carotid sinus and consequently elevates the arterial blood pressure (aBP) [29]. Uğraş and Aksoy [28] also stated that ICP was significantly higher in OS compared with CS and reported a positive correlation between ICP and PaCO_2_ value. ES causes some cardiovascular side effects and may impair cerebral hemodynamic; these effects are worse if patients are disconnected from the ventilator in OS than if they remain connected to the ventilator during CS [30]. Thus, regarding the adverse effects of PaCO_2_ increase on BP and ICP, CS technique appears be safer in patients under OHS.

In this study, SaO_2_ decreased significantly one, five, and fifteen minutes after both techniques, and this decrease was greater in the OS technique. Cereda et al. [10] also found that SaO_2_ decrease after the OS technique was much greater than after the CS technique. Zolfaghari et al. [20] reported that SaO_2_ decrease two and five minutes after the OS technique was greater than that of the CS technique. Mazhari et al. [31] also stated that the OS technique caused a greater decrease in SaO_2_ at different measurement times after suctioning compared to the CS technique.

The patient does not need to be disconnected from the ventilator during CS, and the system maintains constant oxygenation during the procedure; this maintains the lung volume and may explain why SaO_2_ is higher during CS [23, 25]. Given the importance of SaO_2_ normality in OHS patients [17], it seems that CS is more proper than OS.

## 5. Conclusion

This study was conducted to compare the effects of open and closed endotracheal suctioning techniques on the ABG values in patients undergoing open-heart surgery. The results showed that though the variations of PaO_2_, PaCO_2_, and SaO_2_ after endotracheal suctioning were in the normal range, such variations were smaller in closed technique than in open technique. Therefore, regarding the importance of maintaining stability in the hemodynamic status and ABG parameters in patients under OHS, our findings suggest that CS may be superior to OS in patients who have undergone OHS in order to prevent procedure-related complications, especially hypoxemia and hypercapnia following suctioning.

## Figures and Tables

**Table 1:** Demographic characteristics (sex, age group) in two groups (OS and CS) in initial grouping.

Variables	Groups			
	OS number (%)	CS number (%)	Total number (%)	*P* value^*∗*^
Sex				
Male	12 (44.4)	15 (55.6)	27 (64.3)	*P* = 0.333
Female	9 (60.0)	6 (40.0)	15 (23.7)	
Age group (year)				
30–39	1 (100.0)	0 (0.0)	1 (2.4)	*P* = 0.599
40–49	2 (66.7)	1 (33.3)	3 (7.1)	
50–59	7 (58.3)	5 (41.7)	12 (28.6)	
60–69	6 (40)	9 (60)	15 (35.7)	
≥70	5 (45.5)	6 (54.5)	11 (26.2)	
Total	21 (100)	21 (100)	42 (100)	

^*∗*^The results of the chi square test.

**Table 2:** ABG indices in the OS and CS techniques at four measurement times.

ABG indices	Technique	Time				
		Before suction (T0)	1 min after suction (T1)	5 min after suction (T2)	15 min after suction (T3)	*P* value^*∗∗*^
PaO_2_	Open	103.91 ± 8.27	114.44 ± 9.47	98.45 ± 7.94	100.10 ± 7.46	<0.001
	Closed	104.49 ± 7.38	107.50 ± 7.56	103.97 ± 7.53	104.27 ± 7.66	<0.001
	*P* value^*∗*^	0.67	<0.001	<0.001	0.002	—

PaCO_2_	Open	38.21 ± 3.44	39.54 ± 3.80	40.55 ± 4.02	39.13 ± 4.13	<0.001
	Closed	38.46 ± 3.19	36.95 ± 3.09	36.81 ± 3.18	36.81 ± 3.18	<0.001
	*P* value^*∗*^	0.61	<0.001	<0.001	<0.001	—

SaO_2_	Open	96.25 ± 1.88	97.22 ± 1.88	94.17 ± 3.09	94.77 ± 2.74	<0.001
	Closed	96.13 ± 2.11	96.44 ± 2.04	95.89 ± 2.21	96.04 ± 2.01	<0.001
	*P* value^*∗*^	0.60	<0.001	<0.001	<0.001	—

^*∗*^The results of the Wilcoxon test.

^*∗∗*^The results of Freidman's rank test.

## Recommendations for Further Studies

We conducted the study on patients undergoing open-heart surgery. Similar studies are recommended to be carried out on patients with respiratory problems. Moreover, we recommend studies with larger sample size and with more than one time of open and closed suctioning. With respect to the limitations, performing more of the same studies is also suggested with using double-blind methods to control researcher biases, in addition to more studies onto clinical measures such as duration of mechanical ventilation, incidence of pneumonia, ICU or hospital length of stay, and cardiac complications.

## Study Limitations

There are many restraints to the study; first, all suction episodes were performed by one member who may deal with significant observer bias compromising the internal validity of the study. Second, regarding the suction events which were not performed by treating bedside clinicians, this may limit the external validity/generalizability of the study. Third, the study is limited by measuring just certain values (PaO_2_, PaCO_2_, and SaO_2_), while these results may not be clinically significant in terms of the factors such as differences in duration of mechanical ventilation, incidence of pneumonia, ICU or hospital length of stay, and cardiac complications; furthermore there were statistically significant differences between the OS and CS groups in physiologic metrics, but all these differences are in normal ranges which may not be clinically significant. Sampling in the initial recruitment was performed through convenience sampling technique and randomization was not done. Also, the confounding variables were unknown, so, regarding the patients who were considered as own control, these limitations have been reduced. Due to the experimental nature of the study, some biases in the selection of subjects and detection of cases may have occurred. However, as the study sample consisted of patients undergoing elective CABG, it does not seem to reflect the heterogeneity of a general ICU, which may consequently reduce the generalizability of the findings of the study.
